# Morphology of External Genitalia in the Genus *Acanthoponera* Mayr, with Redescription of *A. mucronata* (Roger) Male (Hymenoptera: Formicidae: Ectatomminae)

**DOI:** 10.3390/insects16040436

**Published:** 2025-04-21

**Authors:** Stefano Cantone, Andrea Di Giulio

**Affiliations:** 1 Department of Science, University ‘Roma Tre’, 00146 Rome, Italy; stefano.cantone@crea.gov.it; 2 National Biodiversity Future Center (NBFC), 90133 Palermo, Italy; 3 CREA, Research Centre for Olive, Fruit and Citrus Crops, 95024 Acireale, Italy; 4 Interdepartmental Lab of Electron Microscopy (L.I.M.E.), 00146 Rome, Italy

**Keywords:** antennal cleaning, male ants, SEM, volsella sensorium, winged ants

## Abstract

In this study, using scanning electron microscope (SEM) and optical microscopy, we give a detailed description of the *Acanthoponera mucronata* male, supplementing the former male-based genus diagnoses. In particular, we described for the first time the following characters: the morphology of the external genitalia, the peculiar antennal cleaning and the absence of the metapleural gland orifice. In addition, we show the pretarsal claws and the ventral excavation in the gaster that represent diagnostic male features of all *Acanthoponera* species, never imaged before. The use of modern taxonomic standards is particularly important in order to make these data available to future comparative analyses.

## Simple Summary

In this work, we describe the male *Acanthoponera mucronata* using scanning electron microscopy (SEM) and optical microscopy. We present new findings, including the morphology of the external genitalia, the antennal cleaning, and the absence of the metapleural gland orifice.

## 1. Introduction

The Neotropical ant genus *Acanthoponera* Mayr 1862, included in the subfamily Ectatomminae [1], is distributed from southern Mexico to northern Argentina [2]. The four species of this genus, *A. goeldii* Forel, 1912, *A. minor* (Forel, 1899), *A. mucronata* (Roger, 1860) and *A. peruviana* Brown, 1958, are arboreal, predatory, nocturnal and with little known biology [2,3,4,5]. The taxonomy of this genus is mainly based on the workers’ morphology, while knowledge on males is still scarce [4,6,7].

The morphological diagnosis of males of the genus *Acanthoponera* was initially made by Emery [6]. Later, Brown [7] published a more accurate diagnosis of the male morphological characters, while Kusnezov [8] showed a drawing of the hindwing of the *A. mucronata* male. A more exhaustive description of the genus *Acanthoponera*, with a male-based diagnosis, was made by Feitosa [4], with first description of the *A. mucronata* male and commenting as misidentification the male description of Ketterl and Verhaagh [9]. Recently some hints of male ectathomorph (formerly Ectatomminae + Heteroponerinae) were presented by Boudinot [10], and Cantone [11,12] proposed a brief male morphological diagnosis of the genus *Acanthoponera*; moreover, Cantone and Von Zuben [13] showed a picture of male hindwing. However, in all the above cited morphological descriptions, the male external genitalia have never been studied or illustrated.

In this study we used scanning electron (SEM) and light microscopy to describe and illustrate the male distinctive morphology of the species *A. mucronata*, type species of the genus, and for first time show the morphology of the external genitalia and other important diagnostic features, contributing to a more exhaustive morphological diagnosis of males of the genus *Acanthoponera*.

## 2. Material and Methods

Thirteen male specimens of *Acanthoponera mucronata* were collected in February 2014 in the city of São Paulo, Brazil (23°27′33″ S, 46°38′17″ W, 900 m sea level) using light traps equipped with ultraviolet black blue lamps. Voucher individuals are deposited in the entomological collections of the Department of Science, Roma Tre University (Rome, Italy) and 4 male specimens were donated to the Museum of Zoology University of São Paulo (São Paulo, Brazil).

Taxonomic identification was performed following the diagnosis made by Brown [7] and Feitosa [4].

### 2.1. Morphological Analysis

The identifications and dissections were performed by using Leica MZ12 (Leica Microsystems, Wetzlar, Germany) and Olympus SZX16 (Olympus, Tokyo, Japan) stereomicroscopes equipped respectively with Olympus Highlight 2100 and Olympus KL1500 LCD strong fiber optics. Dissected specimens were mounted on slides in Canada balsam and analyzed with Olympus BX51 (Olympus, Tokyo, Japan) microscope. Specimens were imaged with a Zeiss Axio Zoom V16 (Carl Zeiss AG; Oberkochen, Germany) equipped with an Axiocam 503 (Carl Zeiss Microimaging Gmbh, Jena, Germany) and a Led dual spot lights Photonic Optische (Vienna, Austria).

The Scanning Electron Microscopy analysis was performed at L.I.M.E. Lab. (University of Roma Tre, Rome, Italy). Samples were dehydrated in a graded ethanol series (70%, 85%, 95%, 30 min each and 100% for 2 h), critical point-dried (Balzer Union CPD 030 unit), mounted on aluminum stubs with a conductive adhesive carbon disk, sputtered with a thin layer (30 nm) of gold in a Emithech K550 sputter coater (Emithech, Kent, UK), and analyzed with a Zeiss Gemini 300 field emission SEM microscope at a voltage of 5 kV (Carl Zeiss AG, Jena, Germany).

The descriptions in this work is based on the following morphological studies: general morphology: Yoshimura & Fisher [14,15], Keller [16]; Boudinot, [10], Delsine et al. [17]; antenna cleaner: Schönitzer and Lawitzky [18], Francoeur and Loiselle [19], Beutel et al. [20]; external genitalia: Snodgrass [21,22], Forbes J. and Hagopian M. [23], Boudinot [24], Cantone and Di Giulio [25,26]; wings: Dlussky et al. [27], Perfiliveva [28], Cantone [11,12], Cantone and Von Zuben [13]; head: Richter et al. [29,30]; cuticule microsculpturing: Hellenbrand and Penik [31]; metasoma: Lieberman et al. [32].

### 2.2. Measurements

Measurements reported in this paper were based on the 4 specimens.

The following acronyms were used in the descriptions:

HL: Head length, in full face view. The midline distance from the level of the maximum posterior projection of the margin of the head (not including the ocelli) to the level of the most anterior projection of the anterior clypeal margin.

HW: Head width, in full face view, the maximum width of the head posterior to the compound eyes.

SL: Antennal scape length, measured from the apex of the first antennal segment to the base, exclusive of the radicle.

EL: Eye length, in full face view, the length of the compound eye along the longitudinal axis.

EW: Eye width, with eye held in focal plane facing the viewer, the maximum transverse width of the compound eye.

MML: Maximum mesosomal length.

WL: Forewing length, the maximum distance between the insertion of the sclerotized wing veins to the distal margin of the wing.

WHL: Hindwing length, the maximum distance between the insertion of the sclerotized wing veins to the distal margin of the wing.

CI: Cephalic index. 100× HW/HL.

SI: Scape index. 100× SL/HL.

OI: Ocular index. 100× EL/HL.

WI: Wing index (based on the forewings only). 10× WL/MML.

## 3. Results

### 3.1. Male-Based Morphological Diagnosis of the Genus Acanthoponera

Antennae filiform of 13 articles, scape shorter than second article of the funiculus. Mandibles triangular, dentate. Notauli present. Forewing with submarginal 1 and 2 cell, discoidal cell with 2 M vein present and marginal cell closed; cross-vein 2r-rs and rs-m in line. Hindwing with 2 M vein present and jugal lobe absent. Antennal cleaning with velum without calcar comb. Metatibial spur short and pectinate. Pretarsal claw with large basal lobe and wide preapical and apical tooth. Petiole with anterior face convex and inclined posteriorly with dorsal short lobe, and ventral process developed. Abdominal sternite V, VI, VII and VIII medially concave with tufts of long setae laterally; abdominal tergites with scattered erected setae posteriorly. External genitalia: paramere with lobate telomere; volsella with lobate cuspis, falcate digitus with apicodorsal process and volsella sensorium; valviceps lamina dentate.

### 3.2. Acanthoponera mucronata ♂ (Roger, 1860)

Measurements (in mm; n: 4): HL: 1.23–1.27; HW: 1.21–1.25; SL: 0.22–0.30; EL: 0.65–0.68; EW: 0.39–0.41; MML: 3.16–3.18; WL: 6.57–6.59; WHL: 5.13–5.16.

Indices: ES: 26.8; CI: 98.4; SI: 20.8; OI: 53.6; WI: 20.7.

**Figure 1:**
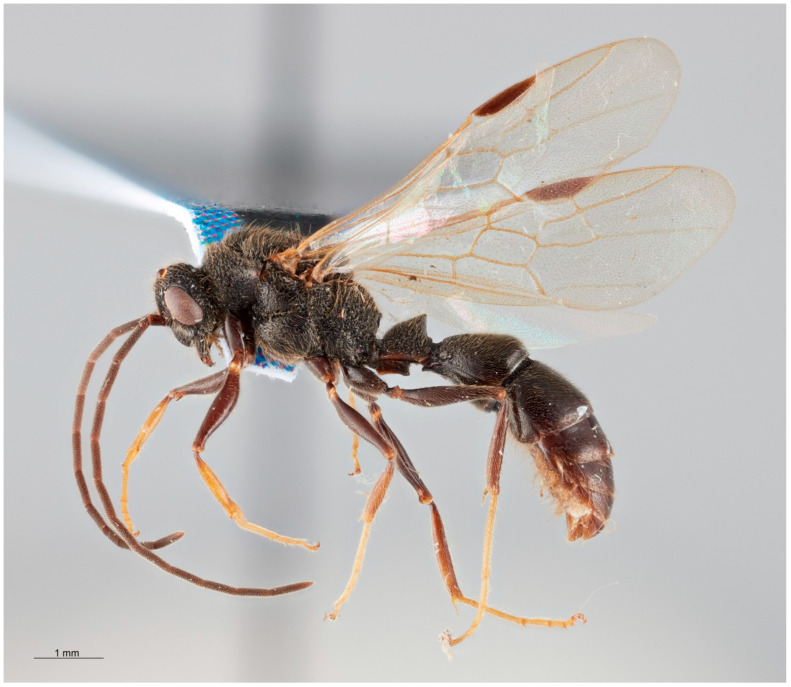
*Acanthoponera mucronata* ♂, left lateral habitus.

Habitus (Figure 1): Color black with tibiae and tarsi deep yellow. Head, mesosoma and petiole with punctate sculpture, erected and decumbent setae. Metasoma smooth and with tufts of long setae laterally.

**Figure 2:**
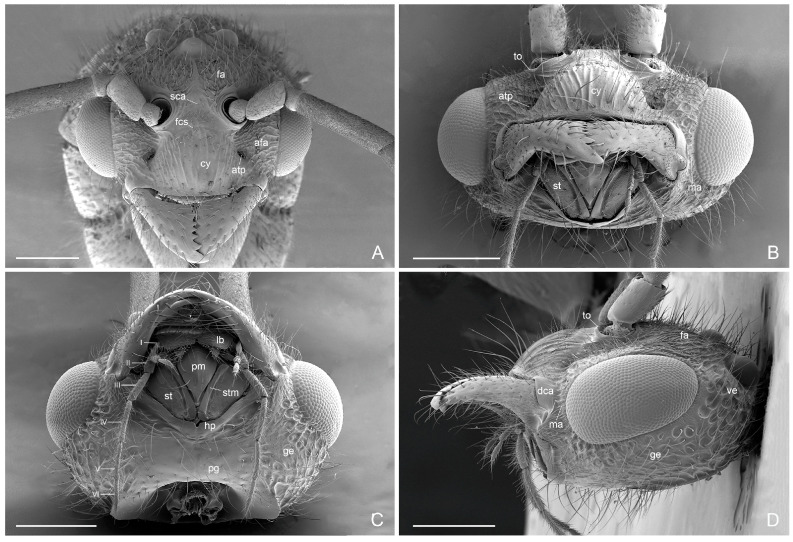
*Acanthoponera mucronata* ♂ (**A**): head in dorsal view; (**B**): head frontal view; (**C**): head ventral view; (**D**): head in lateral view. afa: anterior frontal area; atp: anterior tentorial pit; cy: clypeus; fa: frontal area; fcs: frontoclypeal sulcus; ge: gena; hp: hypostoma; lb: labrum; ma: malar area; pg: postgena; pm: prementum; sca: supraclypeal area; st: stipes; stm: inner stipital margin; to: torulus; ve: vertex; I–VI: articles maxillar palp. Scale-bar: (**A**–**D**) = 400 µm.

Head (Figure 2A–D): Mandibles armed with six teeth on the masticatory margin, apical tooth longest and more robust than others. Maxillary palps with six articles: I very short, II widest, IV, V and VI thinner and longer; and labial palps with for articles. Malar region very short with irregular longitudinal lines. Clypeus strongly convex medially with marked longitudinal subparallel lines and anterior margin straight. Supraclypeal area smooth. Anterior tentorial pit very large. Dorsal clypeal mandibular articulation smooth. Antennae very long, filiform of 13 articles; scape smaller than half second article of the funiculus; first article of the funiculus half of the scape; articles 3–11 of the funiculus subequal, last article longer than the previous ones. Frontal area with irregular longitudinal lines and copious erected setae, anterior frontal area with trasverse irregular lines between the eyes, torulus and anterior tentorial pit. Large elliptical convex eyes in the anterolateral side of the head without setae intraommatidae. Gena and vertex with punctate sculptures. Postgena smooth, with many long scattered setae and hypostoma with small medial notch. Three ocelli present.

**Figure 3:**
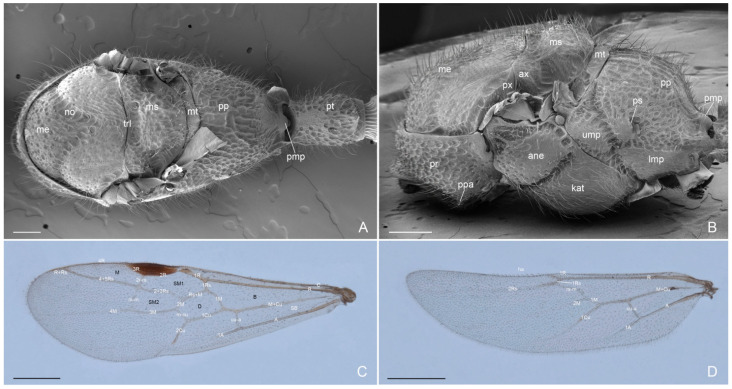
*Acanthoponera mucronata* ♂ (**A**): mesosoma in dorsal view; (**B**): mesosoma in lateral view; (**C**): forewing; (**D**): hindwing. ane: anepisternum; kat: katepisternum; lmp: lower metapleura; me: mesonotum; ms: mesoscutellum; mt: metanotum; pp: propodeum; pmp: propodeal median process; pr: pronotum; ps: propodeal spiracle; ppa: pronotal projection anterolateral; pt: petiole; ump: upper metapleura. A: anal vein; B: basal cell; C: costa vein; Cu: cubital vein; cu-a: cubitus-anal cross-vein; D: discoidal cell; ha: hamuli; M: marginal cell; 1 M: media vein; M + Cu: media + cubitus vein; m-cu: media-cubitus cross-vein; R: Radius vein; Rs: Radial sector vein; rs-m: radial sector-media cross-vein; R + Rs: radius + radial sector vein; r-rs: radial-radial sector cross-vein; SB: subbasal cell; Sc + R: subcosta + radial vein; SM: submarginal cell. Scale-bar: (**A**) = 300 µm, (**B**) = 400 µm, (**C**,**D**) = 1 mm.

**Figure 4:**
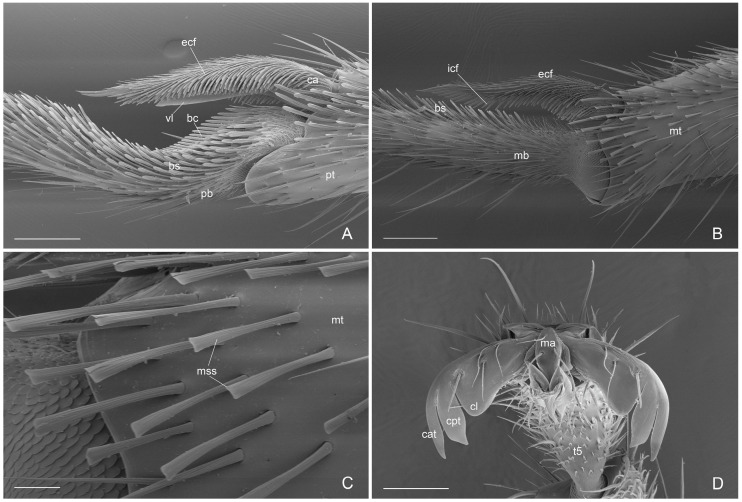
*Acanthoponera mucronata* ♂ (**A**): antennal cleaning; (**B**): metatibial spur; (**C**): metatibial spatulate setae; (**D**): pretarsal claw. bc: basitarsal comb; bs: basitarsal spatulate setae; ca: calcar; cat: claw apical tooth; cl: claw basal lobe; cpt: claw preapical tooth; ecf: external cuticular fringes; icf: internal cuticular fringes; ma: manubrium; mb: metabasitarsus; mss: metatibia spatulate setae; mt: metatibiae; pb: probasitarsus; pt: protibiae; t5: tarsomere 5; vl: velum. Scale-bar: (**A**,**B**) = 100 µm, (**C**) = 20 µm, (**D**) = 60 µm.

**Figure 5:**
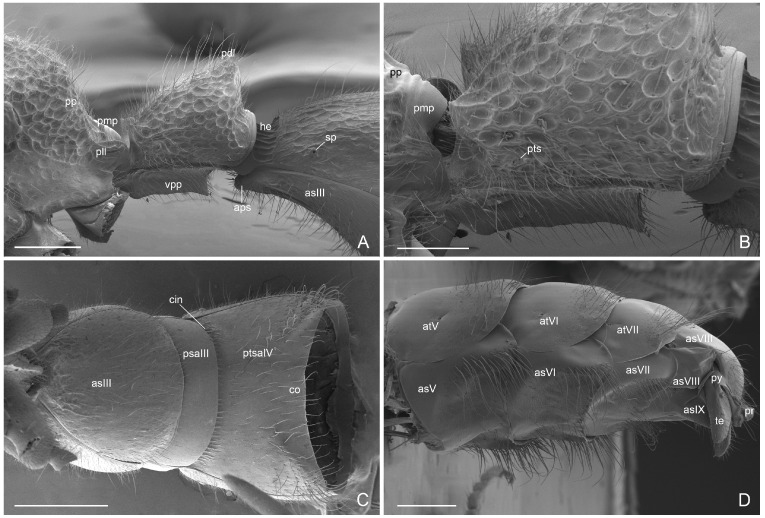
*Acanthoponera mucronata* ♂ (**A**,**B**): Propodeum and petiole in lateral view; (**C**): abdominal sternites III and IV in ventral view; (**D**): abdominal sternites and tergites V, VI, VII, VIII and IX in lateroventral view. as: abdominal sternite; aps: anterior projection abdominal sternite III; at: abdominal tergite; cin: cintus; he: helcium; co: concavity; pdl: petiolar dorsal lobe; pll: propodeum lateral lobe; pmp: propodeum median lobe; pp: propodeum; pr: proctiger; psaIV: abdominal presternite IV; ptsaIV: abdominal poststernite IV; py: pygostyle; sp: abdominal spiracle; te: telomere. Scale-bar: (**A**,**D**) = 400 µm; (**B**) = 200 µm; (**C**) = 300 µm.

Mesosoma (Figure 3A–D, Figure 4A–D and Figure 5A,B): Pronotum, mesonotum, mesoscutellum and propodeum with strongly punctate and reticulate sculpture with abundant erected setae. Pronotum with angulate projection anterolateral. Mesonotum convex with notauli present and strongly engraved. Mesoscutellum in lateral view convex and higher than mesonotum. Metanotum lower than mesoscutellum and at the same height of propodeum. Anepisternum and katepisternum with decumbent setae and superficial punctate sculpture anteriorly, smooth posteriorly. Lower and upper metapleure smooth and punctuate slightly with decumbent setae. Metapleural gland orifice absent. Propodeum convex dorsally and propodeal declivity concave with posterior median process; large and rounded propodeal lobe laterally; propodeal spiracle elliptic and directed posteriorly. Antennal cleaning without comb in the calcar with a low and long velum (or lamella) and external cuticular fringes; probasitarsus with basitarsal comb and spatulate setae. Apex of metatibiae and proximal part of metabasitarsus with spatulate setae, truncate at apex; metatibial spur narrow and pectinate. Pretarsal claws with large basal lobe; preapical tooth very wide and apical tooth longer and less wide, arolium small. Forewing with pterostigma dark, submarginal 1 and 2 cells, discoidal cell with 2 M veins, marginal cell closed; rs-m crossvein in line with 2r-rs crossvein. Hindiwing with 2 M vein, jugal lobe absent and 11–12 hamuli.

Metasoma (Figure 5A–D): Petiole with strongly punctate sculpture dorsally and laterally, with small smooth area dorsoanteriorly; abundant erected and decumbent setae; long and convex anterior surface inclined posteriorly; posterior face short and vertical; apical lobe short; ventral process subrectangular arched posteriorly, with decumbent setae; petiolar spiracle anteriorly. Abdominal tergite III sculptured with irregular line; subsequent tergites smooth with scattered erect setae posteriorly. Abdominal sternites smooth; abdominal sternite III with anterior projection; abdominal segment IV divided by a strong constriction with the sternite slightly concave only posteriorly and with setae ventrolaterally; abdominal sternite V, VI and VII with ventral excavation and tufts of long setae laterally. Short pygostyles. Proctiger poorly developed.

**Figure 6:**
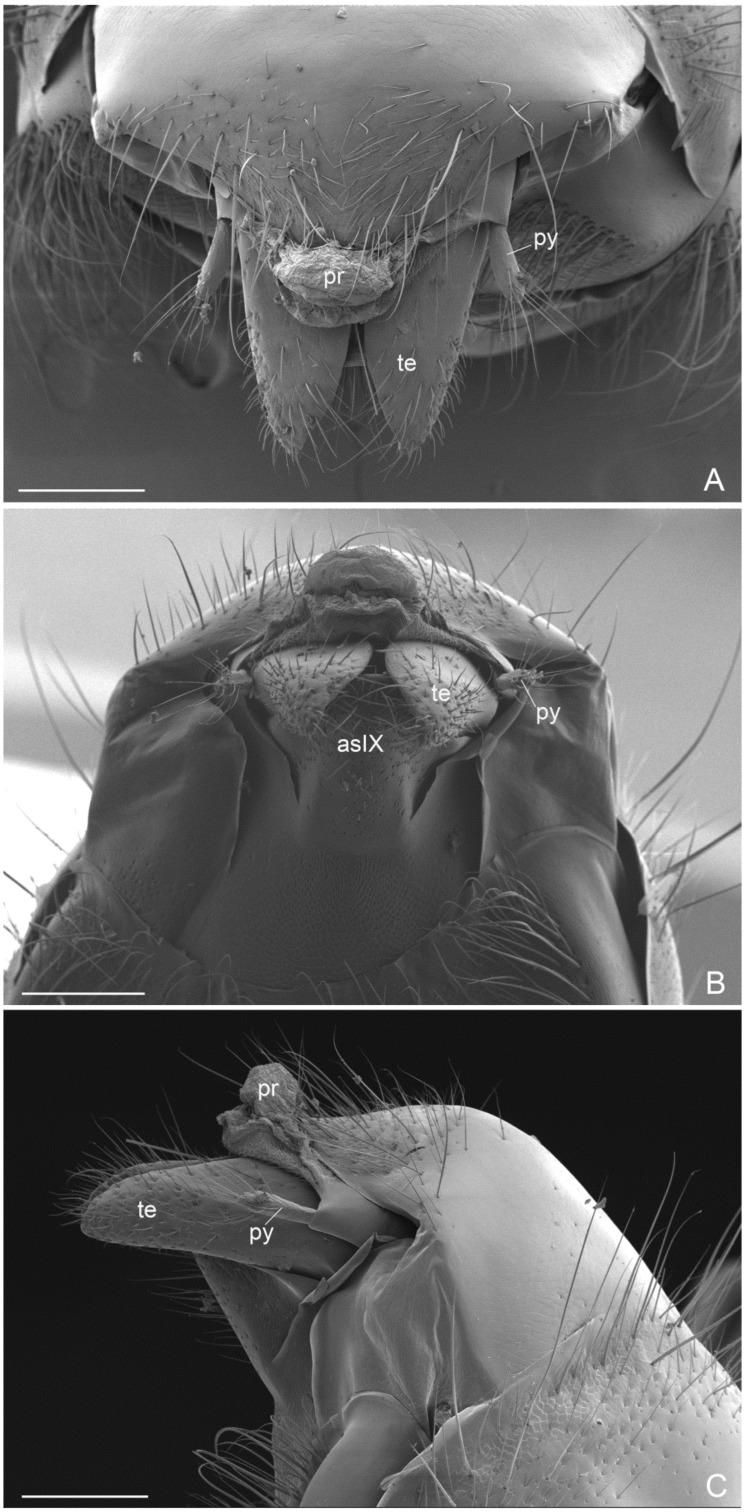
*Acanthoponera mucronata* ♂ External genitalia. (**A**): dorsal view; (**B**): ventral view; (**C**): lateral view. as: abdominal sternite; pr: proctiger; py: pygostyles; te: telomere. Scale-bar: (**A**–**C**) = 200 µm.

**Figure 7:**
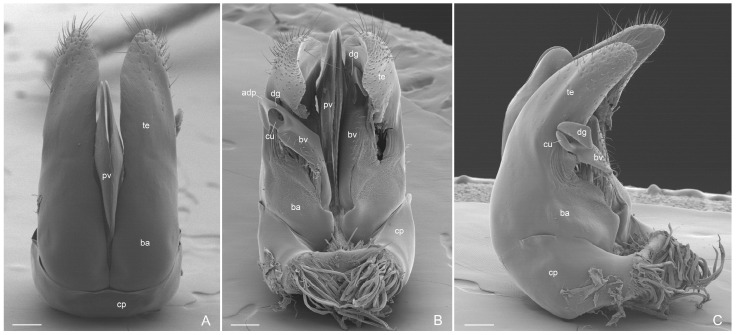
*Acanthoponera mucronata* ♂ External genitalia. (**A**): dorsal view; (**B**): ventral view with left volsella extracted; (**C**): lateroventral view. adp: apicodorsal process; ba: basimere; bv: basivolsella; cp: cupola; cu: cuspis; dg: digitus; pv: penisvalve; te: telomere. Scale-bar: (**A**–**C**) = 100 µm.

**Figure 8:**
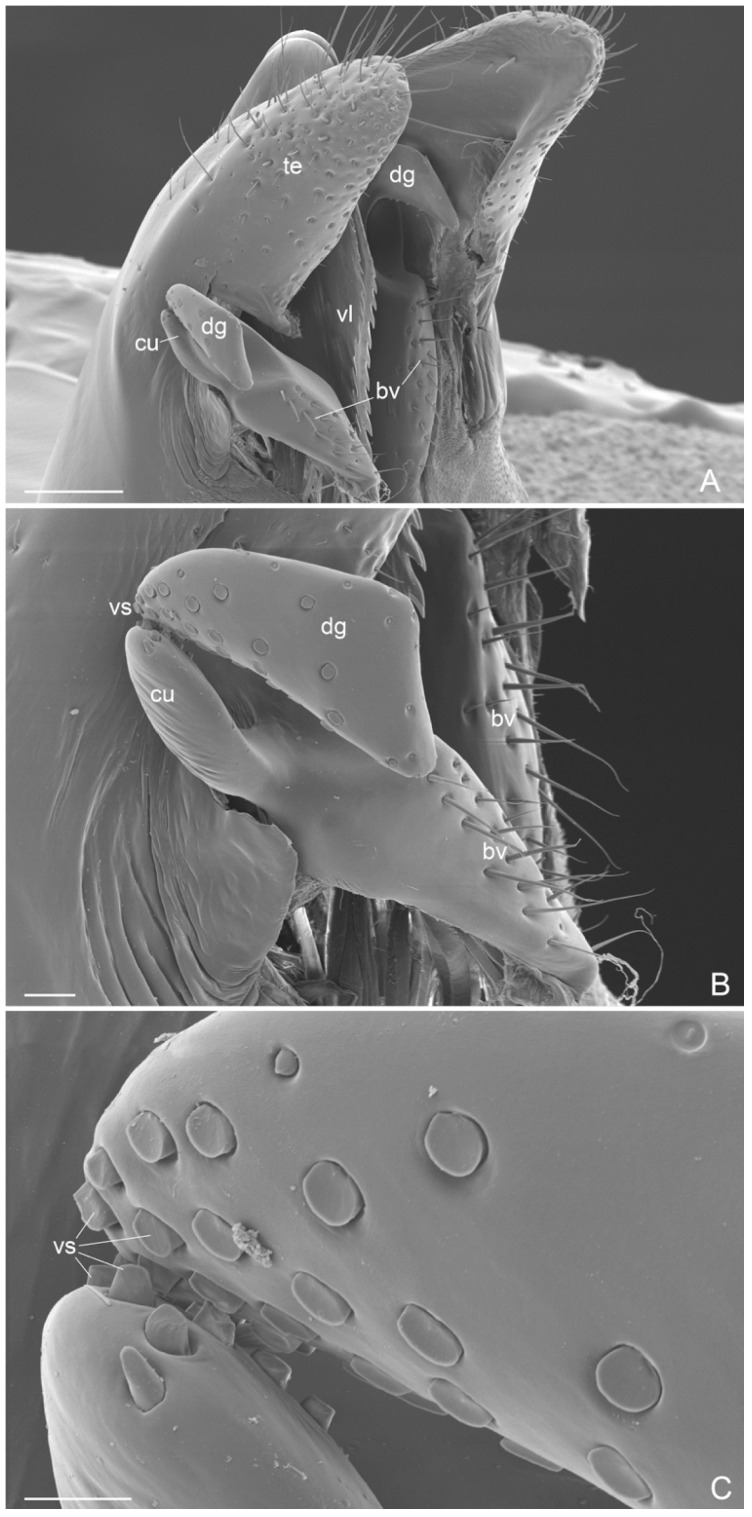
*Acanthoponera mucronata* ♂ External genitalia. (**A**): lateroventral view; (**B**): volsella; (**C**): volsella sensorium. adp: apicodorsal process; bs: button-like sensilla; bvp: basivolsellar process; cu: cuspis; dg: digitus; te: telomere; vs: volsella sensorium; vl: valviceps lamina. Scale-bar: (**A**) = 100 µm, (**B**) = 20 µm, (**C**) = 10 µm.

**Figure 9:**
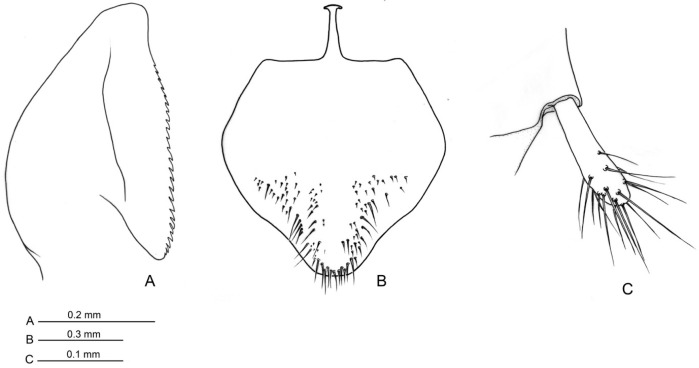
*Acanthoponera mucronata* ♂ (**A**): valviceps lamina; (**B**): IX abdominal sternite; (**C**): left pygostyle, ventral view.

External genitalia (Figure 6A–C, Figure 7A–C, Figure 8A–C and Figure 9A–C): Paramere with basimere developed dorsally and ventrally, telomere lobate; no sulcus trace at junction between basimere and telomere. Volsella: parossiculus with lateral cuspis developed in lobate form with apical and median button-like sensorial sensilla and, ventral basivolsella with long setae; median digitus in falcate form distally with lateral button-like sensilla more numerous on the lateral apicodorsal process; the sensilla in the cuspis and digitus representing the volsella sensorium. Penisvalve without membrane dorsally and with valviceps lamina dentate only anteriorly.

## 4. Discussion

Compared to the previous diagnoses of the genus *Acanthoponera* males [4,7], we give a more detailed description using SEM and light microscopy. We add the first morphological descriptions of the:

(i) antennal cleaning with slender and long velum (lamella) in the calcar, without the calcar comb (Figure 4A). Similar velum morphology is described in some genera of the subfamily Amblyoponinae (*Amblyopone*, *Myopopone, Stigmatomma*); velum of different morphology, characterized by a basal notch, is present in some genera of the subfamilies Ectatomminae (*Rhytidoponera*, *Ectatomma*), Leptanillinae (*Leptanilla*), Ponerinae (*Anochetus*, *Dinoponera*, *Megaponera*, *Odontomachus*, *Pachycondyla*, *Palthothyreus*, *Platythyrea*, *Ponera*, *Pseudoponera*), Paraponerinae (*Paraponera*) and, Pseudomyrmecinae (*Pseudomyrmex*, *Tetraponera*), [16,18,19].
(ii) Absence of the metapleural gland orifice (Figure 3B and Figure 5A). This absence in male of the subfamily Ectatomminae has only been described in *Rhytidoponera metallica* [33,34].
(iii) External genitalia. They show features that can be used for the diagnosis of genus, like: telomere lobiform, presence of a cuspis developed and lobiform and digitus falciform with a apicodorsal process (Figure 6C and Figure 7A). In *A. mucronata* the lateral digitus and the median apical cuspis present the button-like sensilla, which constitute the “volsella sensorium” [26] (Figure 8B,C). The button-like sensilla of the cuspis are in correspondence with some button-like sensilla of the digitus apicodorsal process, showing that they have a mechanosensorial function during mating; in fact, the introduction of the penisvalve is favored by the volsella (cuspis and digitus), which has the function of keeping the last abdominal segments of the female [21,22,24]. In the subfamily Ectatomminae the external genitalia have been described in: *Rhytidioponera metallica* [23], showing similarity of parassiculus structures with *A. mucronata*; *Typhlomyrmex meire* [35]; and in four species of the genus *Ectatomma* [36].
(iv) Apex of metatibiae and proximal part of metabasitarsus with spatulate setae, truncate at apex.

In addition, we show for the first time, with SEM, the metasoma with the characteristic ventral concavity of gaster and the pretarsal claws, which represent distinctive characters of the genus *Acanthoponera* male based.

## 5. Conclusions

This study contributes to knowledge of the male caste of the Neotropical genus *Acanthoponera*, basing on the rare species *A. mucronata* [37]. The use of SEM technique was particularly important for the taxonomic description of the external genitalia, in order to make these data available to future comparative analyses.

## Data Availability

Several male specimens were donated to the Museum of Zoology University of São Paulo (São Paulo, Brazil) for public availability.
